# Dietary Vitamin C Intake Affects Lung Function Through White Blood Cell

**DOI:** 10.1002/fsn3.70299

**Published:** 2025-05-14

**Authors:** Biao Hu, Lu Yuan, Yueyang Zhang, Weiling Deng, Haoyu Zhong, Chengyu Miao, Chudong Wang, Jiaxin Cai

**Affiliations:** ^1^ Department of Radiology The Second Affiliated Hospital of Guangzhou Medical University Guangzhou China; ^2^ Guangzhou Medical University Guangzhou China

**Keywords:** cross‐sectional study, FEV1, FVC, vitamin C, WBC

## Abstract

As an antioxidant, vitamin C has been increasingly used in the treatment of various pulmonary diseases in recent years. However, the mechanism by which vitamin C affects lung function remains unclear to this day. Given its low cost and low risk, vitamin C is highly suitable for widespread use as a conventional treatment, making research into its mechanisms of influencing lung function necessary. Considering the potential association between vitamin C and white blood cells (WBCs), it may influence lung function by affecting white blood count (WBC). The potential impacts of WBCs on the lungs may include damage to the lung parenchyma through proteases released by these cells, as well as the effects of inflammatory factors on alveolar epithelial cells, among other mechanisms. This study aims to explore the potential relationship between dietary vitamin C intake, WBC, and lung function through a cross‐sectional study. This cross‐sectional study included data from 15,738 participants in the National Health and Nutrition Examination Survey (NHANES) from three time periods: 2007–2008, 2009–2010, and 2011–2012. Parallel mediation analysis was conducted using a multivariable logistic regression model to assess the relationships between dietary vitamin C intake, WBC, and lung function. Following the cross‐sectional study, we further incorporated Mendelian randomization (MR) analysis to strengthen the validity of the findings. The results of this cross‐sectional study showed that dietary vitamin C intake was negatively associated with WBC (*p* < 0.05, *β* < 0), while WBC was also negatively associated with lung function. In contrast, dietary vitamin C intake was positively associated with lung function, with a significant positive mediation effect (*p* < 0.05, *β* > 0). These findings suggest that vitamin C may influence lung function by modulating WBC levels. The study may reveal part of the mechanism through which vitamin C affects lung function, specifically through the mediation of WBC. The roles of inflammation and proteases could be potential underlying mechanisms. However, further research is required to clarify the biochemical mechanisms. This study provides a reference for the clinical use of vitamin C in the treatment of related pulmonary diseases and promotes further research into its broader effects.

## Graphical abstract

This study found that dietary vitamin C intake is positively associated with lung function and negatively associated with WBC count, which in turn negatively impacts lung function. Mediation analysis and Mendelian randomization suggest that vitamin C may enhance lung function partially through reducing WBC. These findings highlight a potential anti‐inflammatory mechanism by which vitamin C benefits pulmonary health.

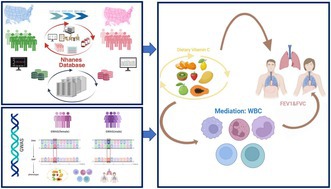

## 1 Introduction

Vitamin C reacts with free radicals and oxidants to form less reactive products, protecting cells from damage during normal physiological processes or diseases. As a widely consumed natural supplement, vitamin C is often considered the preferred antioxidant (Bielski et al. 1975). Vitamin C, as an antioxidant, may theoretically benefit lung function; however, existing research findings on its actual effects remain contradictory, potentially influenced by individual variability and differences in study design. Nonetheless, studies have indicated beneficial effects of vitamin C on lung function, particularly in populations with impaired pulmonary health. A meta‐analysis of 10 randomized controlled trials involving 487 participants demonstrated that vitamin C supplementation (≥ 400 mg/day) significantly improved the percentage of forced expiratory volume in 1 s (FEV1%) in patients with chronic obstructive pulmonary disease (COPD) (Lei et al. 2022). Furthermore, a therapeutic trial targeting elderly pneumonia patients in the United Kingdom reported lower mortality and reduced disease severity in the vitamin C group, while another therapeutic trial conducted in the former Soviet Union on adults across a wide age range observed a dose‐dependent reduction in pneumonia duration with two doses of vitamin C (Hemilä and Louhiala 2013). Among smokers, adequate vitamin C intake has been associated with superior lung function (Shin et al. 2015), and even in pregnant smokers, vitamin C supplementation has been shown to improve neonatal lung function test outcomes (Mcevoy et al. 2014). These findings suggest that vitamin C may exert its effects on lung function through underlying mechanisms. Given the low cost and minimal risk profile of vitamin C, further research into its potential mechanisms remains highly valuable.

Vitamin C concentrations in white blood cells (WBCs) are 50 to 100 times higher than in plasma, enabling it to play a crucial role in cellular functions (Bergsten et al. 1990; Evans et al. 1982). Taking neutrophils as an example, vitamin C supplementation has demonstrated improvements in their chemotactic function among populations with specific pathological conditions. For instance, administering 400 mg/day vitamin C to neonates with suspected sepsis significantly enhanced neutrophil chemotaxis (Vohra et al. 1990). Similarly, patients with chronic granulomatous disease (CGD) showed improved leukocyte chemotaxis following enteral or parenteral vitamin C supplementation (Anderson 1981, 1982). At the molecular level, this vitamin C‐dependent enhancement of chemotaxis may involve improved microtubule assembly through increased stable acetylated α‐tubulin (Boxer, Albertini, et al. 1979; Boxer, Vanderbilt, et al. 1979; Parker et al. 2016), and could also be associated with its antihistamine properties (Johnston et al. 1992). Studies also show that vitamin C reduces reactive oxygen species (ROS) production in WBCs and inhibits the activation of the pro‐inflammatory transcription factor NF‐kB. This helps modulate immune responses and reduce inflammation at the cellular level, as observed in vitro (Carr and Maggini 2017; Mohammed et al. 2013; Tan et al. 2005), suggesting that vitamin C directly influences the sustained production of oxidants and inflammatory mediators. More importantly, It has been shown that apoptosis of neutrophils is significantly promoted by vitamin C through the protection of the caspase‐dependent apoptosis process, which is highly sensitive to oxidative stress (Carr and Maggini 2017). These results imply that vitamin C could influence WBCs, thereby impacting lung function.

To assess lung function, FEV1 and FVC are commonly used as representative indicators. Some studies have indicated that WBC is negatively correlated with both FVC and FEV1, and WBC can damage lung parenchyma through the release of proteases (Wu et al. 2021), such as serine protease and cysteine protease secreted by neutrophils (Owen 2008), and eosinophil peroxidase secreted by activated eosinophils (Mukherjee et al. 2018), which ultimately impair lung function. These observations highlight the damaging effect of WBC on lung function.

However, the specific mechanism by which vitamin C affects lung function has not been fully explained. Therefore, to explore whether vitamin C influences lung function through its effect on WBC and whether WBC mediate the relationship between vitamin C and lung function, this study uses data from the NHANES database (Ahluwalia et al. 2016).

## 2 Methods

### 2.1 Cross‐Sectional Study

#### 2.1.1 Study Population

**FIGURE 1:**
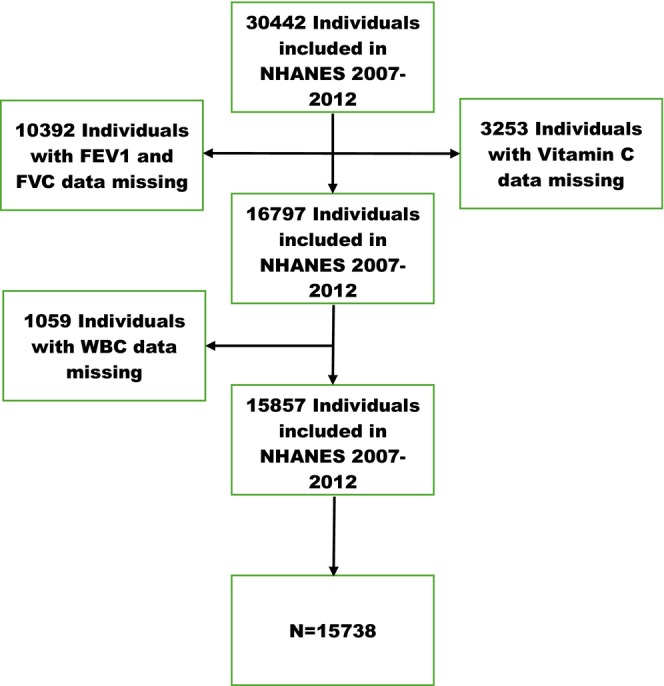
Flowchart illustrating participant selection for this study. *N* = 15,738, NHANES.

Data from the NHANES 2007–2008, 2009–2010, and 2011–2012 (NHANES Questionnaires, Datasets, and Related Documentation) are analyzed by us, including a total of 30,442 participants. After excluding 10,392 individuals with unknown FVC and FEV1, 3253 with unknown dietary vitamin C intake, and 1059 with unknown WBC, 15,738 participants remained. There were 15,738 participants that are included in our study (Figure 1).

#### 2.1.2 Dietary Vitamin C Intake

All NHANES participants underwent two 24‐h dietary recall interviews. The first interview took place at the Mobile Examination Center (MEC), while the second interview was conducted by telephone 3 to 10 days afterward (Zipf et al. 2013). For the purposes of this study, we grouped the data from the MEC interview as DAY1 (D1), and the data from the follow‐up telephone interview as DAY2 (D2). The average dietary vitamin C intake was then calculated by averaging the data from both days.

#### 2.1.3 WBC

The complete blood count (CBC) parameters were derived using Beckman Coulter's counting and quantification methods, which incorporated automatic dilution and mixing devices for sample processing, along with single‐beam spectrophotometry for hemoglobin measurement. WBC differentiation was performed using VCS technology. The quality control and quality assurance (QA/QC) protocols of NHANES follow the standards set by the 1988 Clinical Laboratory Improvement Amendments (CLIA) (Zipf et al. 2013). We use these data and get corresponding WBC.

#### 2.1.4 FEV1 and FVC

Spirometry testing was carried out until the participant successfully completed at least three acceptable maneuvers, reached a maximum of eight spirometry curves, or could no longer proceed. The objective was to complete three acceptable exhalation maneuvers in compliance with ATS standards. FVC and FEV1 were derived from the two highest values obtained from acceptable forced expiratory maneuvers, ensuring minimal variability (e.g., the two highest FVC values should be within 150 mL of each other) (Zipf et al. 2013). Our study's final FEV1 and FVC values were the averages of the two maximum values.

#### 2.1.5 Covariates

Many factors can influence lung function, so we adjusted for potential confounders. Smoking significantly impacts lung function and is a major cause of COPD (Lugg et al. 2022). Alcohol consumption also increases the risk of pulmonary diseases (Ochoa et al. 2022). Hypertension and diabetes mellitus (DM) are other factors that could affect lung function (Schnabel et al. 2011; James 2024). Income, which can influence dietary quality, may also impact lung function (Munro et al. 2023). Therefore, adjustments were made for potential confounders to ensure the reliability of the final data.

Race and ethnicity categories include Mexican‐Americans, non‐Hispanic blacks, non‐Hispanic whites, other Hispanics, and other races, including multiracial individuals. Marital status is categorized as married or unmarried, with the married group including cohabitation, separation, divorce, and widowhood. Smoking status is categorized into three groups: current smokers, former smokers, and never smokers. Participants who have never smoked 100 cigarettes in their lifetime are classified as never smokers. Those who have smoked more than 100 cigarettes but no longer smoke are considered former smokers. Individuals who have smoked over 100 cigarettes and currently smoke, whether occasionally or daily, are categorized as current smokers. Household income is evaluated using the poverty income ratio (PIR), which is calculated based on a specific threshold that takes household size into account. This allows for a more accurate assessment of income relative to the number of people in a household. In terms of health indicators, body mass index (BMI) is widely recognized as a key measure of obesity and overall health, derived from an individual's weight and height. It provides a straightforward way to categorize individuals into different weight groups, such as underweight, normal weight, overweight, and obese. Regarding physical activity, it is classified into three categories: none or unknown, moderate, or vigorous. Moderate physical activity results in a slight increase in respiration or heart rate, such as brisk walking or cycling, while vigorous activity leads to a significant rise in respiration or heart rate, like running or intense sports. These classifications help in understanding various health aspects, such as economic status, obesity risk, and physical activity levels, which can influence overall well‐being. Hypertension or diabetes is diagnosed if any of the following criteria are met. For diabetes, the criteria are as follows: (1) a doctor has diagnosed the participant with diabetes, (2) self‐reported long‐standing diabetes, (3) HbA1c > 6.5%, (4) fasting glucose ≥ 7.0 mmol/L, (5) random blood glucose ≥ 11.1 mmol/L, (6) 2‐h OGTT blood glucose ≥ 11.1 mmol/L, (7) use of diabetes medication or insulin, or (8) being diagnosed with diabetes at birth (type 1 diabetes). Hypertension is diagnosed according to the standards set by the International Society of Hypertension, as well as based on responses from a self‐reported questionnaire. A participant is considered hypertensive if they meet any of the following: (1) current use of antihypertensive medication, (2) a physician's accurate diagnosis, (3) real‐time blood pressure measurements ≥ 140/90 mmHg, (4) self‐reported prior diagnosis of hypertension and current use of blood pressure‐lowering medication, or (5) ambulatory blood pressure monitoring (ABPM) criteria: mean blood pressure ≥ 130/80 mmHg over 24 h, ≥ 135/85 mmHg during the day, and ≥ 120/70 mmHg at night. Data on alcohol consumption were collected through a questionnaire, with participants classified as alcohol drinkers (those consuming at least 12 alcoholic beverages annually) or non‐drinkers (those consuming fewer than 12 alcoholic beverages per year).

In a word, we included baseline data of the population and some conventional indicators as covariates. All results in Table 2 are adjusted for the year circle, age, gender, race, marriage status, PIR, BMI, smoking status, DM, Hypertension, Drinking status, and physical activity. By incorporating covariates into the regression equation, we can control for confounding factors. The NHANES database is a database of normal healthy people, and there are not many basic diseases. For common diseases, hypertension and diabetes, we have included regression equations in the form of covariates. For our inclusion criteria, we did not exclude individuals with unknown covariate indicators. We retained these individuals and categorized their other indicators as a variable labeled “No/unknown.”

#### 2.1.6 Statistical Analysis

##### 2.1.6.1 Multivariable Linear Regression‐Based Mediation Analysis

**FIGURE 2:**
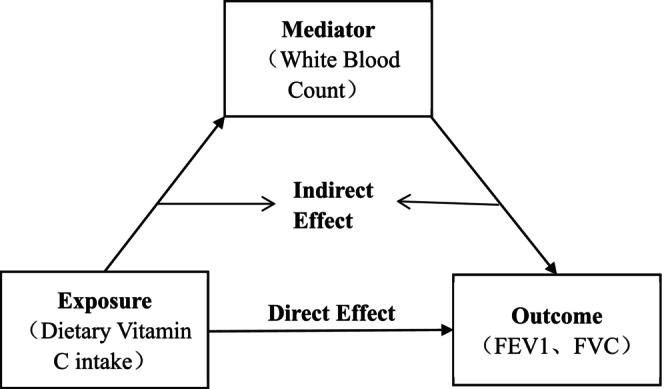
Workflow of Multivariable Linear Regression‐Based Mediation Analysis.

To explore whether WBC mediates the link between dietary vitamin C intake and lung function (FEV1, FVC), we performed a parallel mediation analysis using a multivariable logistic regression model (Figure 2), where WBC served as the mediator. The regression model was employed to evaluate the relationships and effects among the various variables. Adjustments were made for year, age, gender, race, marital status, PIR, BMI, smoking status, diabetes, hypertension, alcohol use, and physical activity. The unstandardized regression coefficients were reported, along with standard errors in parentheses. The direct effect (DE) represents the relationship between vitamin C and lung function independent of the mediator, whereas the indirect effect (IE) captures the influence of vitamin C on lung function through the mediation of WBC. The total effect (TE) represents the overall causal effect of vitamin C on lung function. The proportion of the mediation effect by WBC was calculated by dividing IE by TE (Figure 2).

### 2.2 Mendelian Randomization (MR)

**FIGURE 3:**
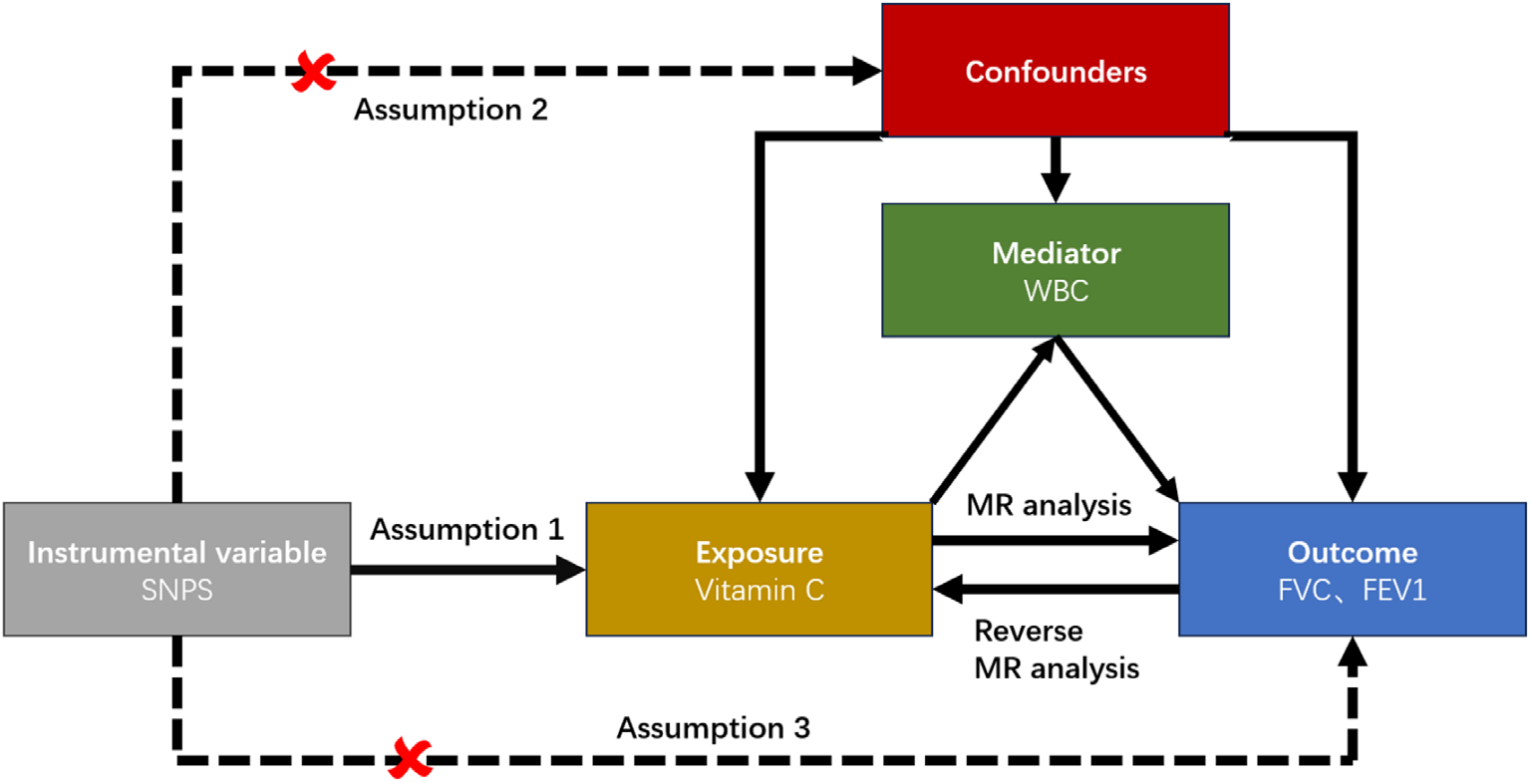
Workflow of Two‐Step Mendelian Randomization Mediation Analysis.

In epidemiological research and analysis, issues such as residual confounding and reverse causality frequently arise. MR can mitigate these biases, thereby generating more robust evidence to clarify which interventions yield health benefits (Davey Smith and Hemani 2014). The principle of MR is rooted in Mendel's second law, which states that genetic alleles segregate independently during gamete formation when DNA is transmitted from parents to offspring (Larsson et al. 2023). Specifically, MR is a statistical method based on three key assumptions: (1) the instrumental variable is strongly associated with the exposure, (2) the instrumental variable is not associated with confounders, and (3) the instrumental variable influences the outcome only through the exposure. By mitigating the impact of residual confounding, MR provides stronger evidence for causal inference than traditional observational studies and, in some cases, even surpasses randomized controlled trials in reliability. This study conducted a univariable MR analysis to further validate the conclusions drawn from the cross‐sectional analysis. The primary MR analysis was performed using the inverse‐variance weighted (IVW) method under a random‐effects model, which combines Wald ratios by dividing the SNP‐outcome effect by the SNP‐exposure effect (Burgess et al. 2013). The IE captures the influence of vitamin C on lung function through the mediation of WBC. The TE represents the overall causal effect of vitamin C on lung function. The proportion of the mediation effect by WBC was calculated by dividing IE by TE (Figure 3). Furthermore, MR's data comes from https://gwas.mrcieu.ac.uk/datasets/, and the select of instrumental variable is in Table S1.

## 3 Results

### 3.1 Cross‐Sectional Study

#### 3.1.1 Participant Demographics

Table 1 shows the demographic characteristics of the 15,738 NHANES participants collected between 2007 and 2012.

**TABLE 1:** Demographic characteristics of participants.

Variables	Total (*n* = 15,738)
Year, *n* (%)	
2007–2008	4922 (31.3)
2009–2010	5596 (35.6)
2011–2012	5220 (33.2)
Age, mean ± SD	36.4 ± 21.2
Gender, *n* (%)	
Female	7912 (50.3)
Male	7826 (49.7)
Race, *n* (%)	
Mexican American	2886 (18.3)
Non‐Hispanic black	3486 (22.2)
Non‐Hispanic white	6323 (40.2)
Other Hispanic	1733 (11.0)
Other race‐including multiracial	1310 (8.3)
Marry, *n* (%)	
Never married	2057 (19.0)
Divorced	1196 (11.1)
Living with partner	889 (8.2)
Married	5753 (53.2)
Separated	358 (3.3)
Widowed	556 (5.1)
PIR, mean ± SD	2.4 ± 1.6
BMI, mean ± SD	26.9 ± 7.4
Smoke, *n* (%)	
Never	5934 (54.9)
Former	2533 (23.4)
Now	2342 (21.7)
DM, *n* (%)	
No/unknown	12,949 (82.3)
Yes	2789 (17.7)
Hypertension, *n* (%)	
No/unknown	10,837 (72.2)
Yes	4180 (27.8)
Alcohol, *n* (%)	
Yes	2725 (25.9)
No/unknown	7808 (74.1)
Physical activity, *n* (%)	
Unknown	8290 (52.7)
Moderate	3421 (21.7)
Vigorous	4027 (25.6)
WBC (10000 cells/μL), mean ± SD	70.5 ± 22.5
FVC (mL), mean ± SD	3636.3 ± 1187.4
FEV1 (mL), mean ± SD	2917.6 ± 957.7
Ave. vitamin C (mg/day), median (IQR)	67.0 (32.4, 116.7)

Abbreviations: BMI, body mass index; DM, diabetes mellitus; IQR, interquartile range; PIR, poverty income ratio.

Data on smoke: never = smoked less than 100 cigarettes in life; former = smoked less than 100 cigarettes in life and smoke not at all now; now = smoked more than 100 cigarettes in life and smoke some days or every day.

Data on alcohol: Yes = at least 12 alcoholic drinks per year; No = less than 12 alcoholic drinks per year.

Data on physical activity: Moderate: In a typical week, engage in moderate‐intensity sports, fitness, or recreational activities—such as brisk walking, cycling, swimming, or volleyball—that result in a slight increase in breathing or heart rate, for at least 10 min without interruption; Vigorous: In a typical week, engage in vigorous‐intensity sports, fitness, or recreational activities—such as running or basketball—that significantly increase breathing or heart rate, for at least 10 min continuously.

#### 3.1.2 Linear Regression Model Results

Table 2 illustrates the associations between dietary vitamin C intake, WBC, and lung function (FEV1, FVC). The results indicate that dietary vitamin C intake was negatively correlated with WBC, and WBC was negatively correlated with lung function. DE indicates that vitamin C has a positive effect on lung function, while IE, also positive, was smaller than DE, suggesting that WBC plays a mediating role in the effect of vitamin C on lung function. The mediation proportion calculated using FEV1 and FVC as outcomes was 6.743% and 7.311%, respectively.

**TABLE 2:** The mediating role of white blood cell count in the relationship between dietary vitamin C intake and lung function (Multivariable Linear Regression‐Based Mediation Analysis from Nhanes database).

Exposure: dietary vitamin C intake (mg/day); mediator: white blood count (10,000 cells/μL); outcome: FEV1, FVC (mL)						
Outcome	Exposure to mediator	Mediator to outcome	Direct effect	Mediated (indirect) effect	Total effect (exposure to outcome)	Proportion mediated (%)
FEV1	−0.0095 (0.0030)**	−1.8120 (0.0705)***	0. 2365 (0.0705)***	0.0171 (0.0065)**	0. 2536 (0.0706)***	6.743
FVC	−0.0095 (0.003)**	−1. 9061 (0.2953)***	0. 2282 (0.0859)**	0. 018 (0.0065)**	0.2462 (0.0861)**	7.311

*Note:* Mediation model: Adjust for year circle, age, gender, race, marriage status, PIR, BMI, smoking status, DM, Hypertension, Drinking status, physical activity. Unstandardized regression coefficients are displayed, with standard errors in parentheses.

* *p* < 0.05.

** *p* < 0.01.

*** *p <* 0.001.

#### 3.1.3 MR Results

Table 3 presents the results of MR analysis investigating the mediating role of WBC count in the relationship between dietary vitamin C intake and lung function. The results show a negative association between vitamin C and WBC (*p* < 0.05), while WBC demonstrates a significant negative correlation with lung function (*p* < 0.001). Additionally, reverse causality had minimal impact, further supporting the reliability of the findings. The IE was positive but smaller in magnitude than the DE, indicating that WBC count partially mediates the influence of vitamin C on lung function. The mediation proportions calculated for FEV1 and FVC as outcomes were 3.94% and 2.19%, respectively. The MR analysis provides robust evidence supporting the results of the multivariate linear regression, thereby enhancing the credibility of the conclusions.

**TABLE 3:** The mediating role of white blood cell count in the relationship between dietary vitamin C intake and lung function (Analyze through two‐step mediated Mendelian randomization).

Exposure: serum vitamin C; mediator: white blood count; outcome: FEV1, FVC						
Outcome	Exposure to mediator (IVW)	Mediator to outcome (IVW)	Outcome to exposure (IVW)	Mediated (indirect) effect	Total effect (exposure to outcome, IVW)	Proportion mediated (%)
FEV1	−0.0528 (0.0246)*	−0. 0587 (0.0079)***	0. 0044 (0.0146)	0. 0030 (0.0014)*	0. 0764 (0.0276)**	3.94
FVC	−0. 0488 (0.0211)*	−0.0333 (0.0065)***	0. 0187 (0.0181)	0. 0016 (0.0008)*	0. 0741 (0.0263)**	2.19

*Note:* *β* value of univariate Mendelian randomization analysis results are displayed, with standard errors in parentheses.

* *p* < 0.05.

** *p* < 0.01.

*** *p* < 0.001.

## 4 Discussion

### 4.1 Interpretation of Results

In this study, we used the NHANES 2007–2012 cross‐sectional data and applied linear regression to examine whether vitamin C could influence lung function through the mediation of WBC. Our analytical results suggest that WBC may mediate the effect of vitamin C on lung function.

Regarding the impact of dietary vitamin C intake on WBC, our results align with the current general consensus (Table 2), showing a negative correlation between the two. This could be because vitamin C promotes the chemotactic function of WBC (Carr and Maggini 2017; Bozonet et al. 2015), enabling them to migrate to infection sites where they perform their function and are then cleared (Lämmermann 2016), rather than circulating aimlessly in the bloodstream. More importantly, vitamin C may also promote the apoptosis of neutrophils, an essential process that helps eliminate oxidant‐sensitive cells (Sharma et al. 2004).

The idea that vitamin C influences lung function through WBC is well‐supported by our data. Table 2 demonstrates a notable negative correlation between WBC levels and lung function. The impact of WBC on lung function is linked to the release of proteases by inflammatory cells, which subsequently cause damage to the lung parenchyma (Wu et al. 2021). Several studies have pointed out that individuals with higher eosinophil counts experience faster FEV1 decline (Hong et al. 2024). Although the specific underlying mechanisms remain unclear, this may be due to damage to the lung parenchyma caused by proteases released from eosinophils (Mukherjee et al. 2018) and substances such as TGF‐β that lead to pulmonary fibrosis (Wynn 2011). Also, aging, reduced FEV1, and a history of bronchitis are associated with increased WBC (James et al. 1999). Neutrophils deficient in vitamin C remain in inflammatory sites in vivo, continually contributing to inflammation rather than being cleared by macrophages in vitro (Vissers and Wilkie 2007). Alveolar macrophages are involved in nearly all the scenarios mentioned above, as they can both initiate and resolve immune responses. A disruption in their homeostasis would severely impact lung function (Lugg et al. 2022). These studies support the finding that high WBC damages lung function (Hong et al. 2024; Zeig‐Owens et al. 2018; Hancox et al. 2018), which aligns with our results.

The DE in our results also shows a significant positive correlation between vitamin C and lung function (FEV1, FVC) (*p* < 0.05, standard error < 0.5). Numerous related studies also provide strong evidence for a positive relationship between vitamin C supplementation and improved lung function (Shaheen et al. 2010). For instance, a daily intake of 400 mg of vitamin C is required for a statistically significant improvement compared to a placebo (Lei et al. 2022). The underlying reason why vitamin C impacts lung function is thought to be its antioxidant properties, which reduce oxidative stress (Barnes 2022). Vitamin C has been used to treat various diseases, including COPD (Lei et al. 2022; Barnes 2022; Romieu and Trenga 2001), and conditions with similar pathophysiological features, such as Acute Respiratory Distress Syndrome (ARDS) (Lei et al. 2022; Boretti and Banik 2020). In our results (Table 2), both DE and IE showed positive correlations (*p* < 0.05; standard error < 0.5), and the direction was consistent. Additionally, the IE was found to be smaller than the DE, indicating that WBC may play a role in the mechanism through which vitamin C influences lung function. The mediation proportions of 6.743% and 7.311% calculated from FEV1 and FVC indicate that other mediating factors or mechanisms could also play a role.

In summary, vitamin C may influence lung function through the mediating role of WBCs, and the underlying mechanisms may include affecting the occurrence and duration of lung inflammation, or controlling protease activity in the lung parenchyma, ultimately impacting lung function.

### 4.2 Strengths and Limitations

The main strength of this study lies in the fact that no previous research has identified WBC as a mediator in the relationship between vitamin C and lung function. This study, through an analytical cross‐sectional approach (Wang and Cheng 2020), offers a novel conclusion based on linear regression, making it an effective method for studying relationships involving three variables.

However, there are several limitations in this study. First, as a cross‐sectional study, it is difficult to draw accurate causal conclusions (Savitz and Wellenius 2023). Second, this study focused on dietary vitamin C intake, which does not directly equate to plasma vitamin C levels, and the difference between the two may introduce some error into the final results (Padayatty et al. 2004; Sauberlich 1994).

## 5 Conclusion

In conclusion, our multivariate regression analysis revealed a statistically significant positive association between dietary vitamin C intake and pulmonary function (FEV1/FVC), with WBC demonstrating partial mediation effects accounting for 6.74% to 7.31% of the total observed association. The MR analysis provided corroborating evidence supporting causal relationships among these three factors. But the mediation model explains only approximately 7% of the observed association, suggesting that other potential mechanisms may be jointly involved, which warrants further investigation through multi‐omics studies.

## Author Contributions

J.C., B.H., L.Y., and C.W.: project design. B.H., L.Y., and C.W.: conception and design. B.H.: collection and assembly of data. Y.Z., W.D., H.Z., and C.M.: wrote the manuscript. All authors: data analysis and interpretation, manuscript writing, and final approval of the manuscript.

## Ethics Statement

The national center for health statistics (NCHS) ethical review committee members shall be examined and approved the NHANES studies involving human participants. All participants provided written informed consent to participate in the study. Mendelian randomization analyses used previously obtained summary data from studies that had proper informed consent and ethics approval. No additional ethical permit was required for the secondary analysis of summary data.

## Consent

The authors have nothing to report.

## Conflicts of Interest

The authors declare no conflicts of interest.

## Supporting information


**Table S1.** Selection and criteria of instrumental variables.

## Data Availability

All data is sourced from public databases, publicly available and downloadable. NHANES has developed a public use dataset, available at https://www.cdc.gov/nchs/nhanes/index.html. The data for Mendelian randomization is sourced from the IEU database (https://gwas.mrcieu.ac.uk/datasets/); The data of Mendelian randomization detailed ID number can be found in Table S1.

## References

[fsn370299-bib-0001] Ahluwalia, N. , J. Dwyer , A. Terry , et al. 2016. “Update on NHANES Dietary Data: Focus on Collection, Release, Analytical Considerations, and Uses to Inform Public Policy.” Advances in Nutrition 7, no. 1: 121–134. 26773020 10.3945/an.115.009258 PMC4717880

[fsn370299-bib-0002] Anderson, R. 1981. “Assessment of Oral Ascorbate in Three Children With Chronic Granulomatous Disease and Defective Neutrophil Motility Over a 2‐Year Period.” Clinical and Experimental Immunology 43, no. 1: 180–188. 6265123 PMC1537134

[fsn370299-bib-0003] Anderson, R. 1982. “Effects of Ascorbate on Normal and Abnormal Leucocyte Functions.” International Journal for Vitamin and Nutrition Research. Supplement 23: 23–34. 6811483

[fsn370299-bib-0004] Barnes, P. J. 2022. “Oxidative Stress in Chronic Obstructive Pulmonary Disease.” Antioxidants 11, no. 5: 965. 35624831 10.3390/antiox11050965 PMC9138026

[fsn370299-bib-0005] Bergsten, P. , G. Amitai , J. Kehrl , et al. 1990. “Millimolar Concentrations of Ascorbic Acid in Purified Human Mononuclear Leukocytes. Depletion and Reaccumulation.” Journal of Biological Chemistry 265, no. 5: 2584–2587. 2303417

[fsn370299-bib-0006] Bielski, B. H. , H. W. Richter , and P. C. Chan . 1975. “Some Properties of the Ascorbate Free Radical.” Annals of the New York Academy of Sciences 258: 231–237. 942 10.1111/j.1749-6632.1975.tb29283.x

[fsn370299-bib-0007] Boretti, A. , and B. K. Banik . 2020. “Intravenous Vitamin C for Reduction of Cytokines Storm in Acute Respiratory Distress Syndrome.” PharmaNutrition 12: 100190. 32322486 10.1016/j.phanu.2020.100190 PMC7172861

[fsn370299-bib-0008] Boxer, L. A. , D. F. Albertini , R. L. Baehner , and J. M. Oliver . 1979. “Impaired Microtubule Assembly and Polymorphonuclear Leucocyte Function in the Chediak‐Higashi Syndrome Correctable by Ascorbic Acid.” British Journal of Haematology 43, no. 2: 207–213. 508630 10.1111/j.1365-2141.1979.tb03743.x

[fsn370299-bib-0009] Boxer, L. A. , B. Vanderbilt , S. Bonsib , R. Jersild , H. H. Yang , and R. L. Baehner . 1979. “Enhancement of Chemotactic Response and Microtubule Assembly in Human Leukocytes by Ascorbic Acid.” Journal of Cellular Physiology 100, no. 1: 119–126. 468916 10.1002/jcp.1041000112

[fsn370299-bib-0010] Bozonet, S. M. , A. C. Carr , J. M. Pullar , and M. C. Vissers . 2015. “Enhanced Human Neutrophil Vitamin C Status, Chemotaxis and Oxidant Generation Following Dietary Supplementation With Vitamin C‐Rich SunGold Kiwifruit.” Nutrients 7, no. 4: 2574–2588. 25912037 10.3390/nu7042574 PMC4425162

[fsn370299-bib-0011] Burgess, S. , A. Butterworth , and S. G. Thompson . 2013. “Mendelian Randomization Analysis With Multiple Genetic Variants Using Summarized Data.” Genetic Epidemiology 37, no. 7: 658–665. 24114802 10.1002/gepi.21758 PMC4377079

[fsn370299-bib-0012] Carr, A. C. , and S. Maggini . 2017. “Vitamin C and Immune Function.” Nutrients 9, no. 11: 1211. 29099763 10.3390/nu9111211 PMC5707683

[fsn370299-bib-0013] Davey Smith, G. , and G. Hemani . 2014. “Mendelian Randomization: Genetic Anchors for Causal Inference in Epidemiological Studies.” Human Molecular Genetics 23, no. R1: R89–R98. 25064373 10.1093/hmg/ddu328 PMC4170722

[fsn370299-bib-0014] Evans, R. M. , L. Currie , and A. Campbell . 1982. “The Distribution of Ascorbic Acid Between Various Cellular Components of Blood, in Normal Individuals, and Its Relation to the Plasma Concentration.” British Journal of Nutrition 47, no. 3: 473–482. 7082619 10.1079/bjn19820059

[fsn370299-bib-0015] Hancox, R. J. , I. D. Pavord , and M. R. Sears . 2018. “Associations Between Blood Eosinophils and Decline in Lung Function Among Adults With and Without Asthma.” European Respiratory Journal 51, no. 4: 1702536. 29563173 10.1183/13993003.02536-2017

[fsn370299-bib-0016] Hemilä, H. , and P. Louhiala . 2013. “Vitamin C for Preventing and Treating Pneumonia.” Cochrane Database of Systematic Reviews 8: Cd005532. 10.1002/14651858.CD005532.pub3 23925826

[fsn370299-bib-0017] Hong, Y. S. , H. Y. Park , S. Ryu , et al. 2024. “The Association of Blood Eosinophil Counts and FEV(1) Decline: A Cohort Study.” European Respiratory Journal 63, no. 5: 2301037. 38636990 10.1183/13993003.01037-2023

[fsn370299-bib-0018] James, A. 2024. “Diabetes and Lung Function: Linked, but How?” Respirology 29, no. 5: 361–362. 38379119 10.1111/resp.14674

[fsn370299-bib-0019] James, A. L. , M. W. Knuiman , M. L. Divitini , et al. 1999. “Associations Between White Blood Cell Count, Lung Function, Respiratory Illness and Mortality: The Busselton Health Study.” European Respiratory Journal 13, no. 5: 1115–1119. 10414413 10.1034/j.1399-3003.1999.13e29.x

[fsn370299-bib-0020] Johnston, C. S. , L. J. Martin , and X. Cai . 1992. “Antihistamine Effect of Supplemental Ascorbic Acid and Neutrophil Chemotaxis.” Journal of the American College of Nutrition 11, no. 2: 172–176. 1578094

[fsn370299-bib-0021] Lämmermann, T. 2016. “In the Eye of the Neutrophil Swarm‐Navigation Signals That Bring Neutrophils Together in Inflamed and Infected Tissues.” Journal of Leukocyte Biology 100, no. 1: 55–63. 26416718 10.1189/jlb.1MR0915-403

[fsn370299-bib-0022] Larsson, S. C. , A. S. Butterworth , and S. Burgess . 2023. “Mendelian Randomization for Cardiovascular Diseases: Principles and Applications.” European Heart Journal 44, no. 47: 4913–4924. 37935836 10.1093/eurheartj/ehad736 PMC10719501

[fsn370299-bib-0023] Lei, T. , T. Lu , H. Yu , et al. 2022. “Efficacy of Vitamin C Supplementation on Chronic Obstructive Pulmonary Disease (COPD): A Systematic Review and Meta‐Analysis.” International Journal of Chronic Obstructive Pulmonary Disease 17: 2201–2216. 36118282 10.2147/COPD.S368645 PMC9473551

[fsn370299-bib-0024] Lugg, S. T. , A. Scott , D. Parekh , B. Naidu , and D. R. Thickett . 2022. “Cigarette Smoke Exposure and Alveolar Macrophages: Mechanisms for Lung Disease.” Thorax 77, no. 1: 94–101. 33986144 10.1136/thoraxjnl-2020-216296 PMC8685655

[fsn370299-bib-0025] Mcevoy, C. T. , D. Schilling , N. Clay , et al. 2014. “Vitamin C Supplementation for Pregnant Smoking Women and Pulmonary Function in Their Newborn Infants: A Randomized Clinical Trial.” JAMA 311, no. 20: 2074–2082. 24838476 10.1001/jama.2014.5217 PMC4296045

[fsn370299-bib-0026] Mohammed, B. M. , B. J. Fisher , D. Kraskauskas , et al. 2013. “Vitamin C: A Novel Regulator of Neutrophil Extracellular Trap Formation.” Nutrients 5, no. 8: 3131–3151. 23939536 10.3390/nu5083131 PMC3775246

[fsn370299-bib-0027] Mukherjee, M. , P. Lacy , and S. Ueki . 2018. “Eosinophil Extracellular Traps and Inflammatory Pathologies‐Untangling the Web!” Frontiers in Immunology 9: 2763. 30534130 10.3389/fimmu.2018.02763 PMC6275237

[fsn370299-bib-0028] Munro, H. M. , D. Yu , W. Zheng , W. J. Blot , Q. Cai , and M. J. Shrubsole . 2023. “Diet Quality and Lung Cancer Incidence in a Low‐Income Population in the United States.” British Journal of Cancer 129, no. 4: 626–635. 37400676 10.1038/s41416-023-02342-7 PMC10421925

[fsn370299-bib-0029] Ochoa, C. A. , C. G. Nissen , D. D. Mosley , et al. 2022. “Aldehyde Trapping by ADX‐102 Is Protective Against Cigarette Smoke and Alcohol Mediated Lung Cell Injury.” Biomolecules 12, no. 3: 393. 35327585 10.3390/biom12030393 PMC8946168

[fsn370299-bib-0030] Owen, C. A. 2008. “Roles for Proteinases in the Pathogenesis of Chronic Obstructive Pulmonary Disease.” International Journal of Chronic Obstructive Pulmonary Disease 3, no. 2: 253–268. 18686734 10.2147/copd.s2089 PMC2629972

[fsn370299-bib-0031] Padayatty, S. J. , H. Sun , Y. Wang , et al. 2004. “Vitamin C Pharmacokinetics: Implications for Oral and Intravenous Use.” Annals of Internal Medicine 140, no. 7: 533–537. 15068981 10.7326/0003-4819-140-7-200404060-00010

[fsn370299-bib-0032] Parker, W. H. , E. M. Rhea , Z. C. Qu , M. R. Hecker , and J. M. May . 2016. “Intracellular Ascorbate Tightens the Endothelial Permeability Barrier Through Epac1 and the Tubulin Cytoskeleton.” American Journal of Physiology‐Cell Physiology 311, no. 4: C652–C662. 27605450 10.1152/ajpcell.00076.2016 PMC5129753

[fsn370299-bib-0033] Romieu, I. , and C. Trenga . 2001. “Diet and Obstructive Lung Diseases.” Epidemiologic Reviews 23, no. 2: 268–287. 12192737 10.1093/oxfordjournals.epirev.a000806

[fsn370299-bib-0034] Sauberlich, H. E. 1994. “Pharmacology of Vitamin C.” Annual Review of Nutrition 14: 371–391. 10.1146/annurev.nu.14.070194.002103 7946525

[fsn370299-bib-0035] Savitz, D. A. , and G. A. Wellenius . 2023. “Can Cross‐Sectional Studies Contribute to Causal Inference? It Depends.” American Journal of Epidemiology 192, no. 4: 514–516. 35231933 10.1093/aje/kwac037

[fsn370299-bib-0036] Schnabel, E. , D. Nowak , S. Brasche , et al. 2011. “Association Between Lung Function, Hypertension and Blood Pressure Medication.” Respiratory Medicine 105, no. 5: 727–733. 21276721 10.1016/j.rmed.2010.12.023

[fsn370299-bib-0037] Shaheen, S. O. , K. A. Jameson , H. E. Syddall , et al. 2010. “The Relationship of Dietary Patterns With Adult Lung Function and COPD.” European Respiratory Journal 36, no. 2: 277–284. 20075056 10.1183/09031936.00114709

[fsn370299-bib-0038] Sharma, P. , S. A. Raghavan , R. Saini , et al. 2004. “Ascorbate‐Mediated Enhancement of Reactive Oxygen Species Generation From Polymorphonuclear Leukocytes: Modulatory Effect of Nitric Oxide.” Journal of Leukocyte Biology 75, no. 6: 1070–1078. 15039465 10.1189/jlb.0903415

[fsn370299-bib-0039] Shin, J. Y. , J. Y. Shim , D. C. Lee , et al. 2015. “Smokers With Adequate Vitamin C Intake Show a Preferable Pulmonary Function Test.” Journal of the American College of Nutrition 34, no. 5: 385–390. 25961759 10.1080/07315724.2014.926152

[fsn370299-bib-0040] Tan, P. H. , P. Sagoo , C. Chan , et al. 2005. “Inhibition of NF‐Kappa B and Oxidative Pathways in Human Dendritic Cells by Antioxidative Vitamins Generates Regulatory T Cells.” Journal of Immunology 174, no. 12: 7633–7644. 10.4049/jimmunol.174.12.7633 15944264

[fsn370299-bib-0041] Vissers, M. C. , and R. P. Wilkie . 2007. “Ascorbate Deficiency Results in Impaired Neutrophil Apoptosis and Clearance and Is Associated With Up‐Regulation of Hypoxia‐Inducible Factor 1alpha.” Journal of Leukocyte Biology 81, no. 5: 1236–1244. 10.1189/jlb.0806541 17264304

[fsn370299-bib-0042] Vohra, K. , A. J. Khan , V. Telang , et al. 1990. “Improvement of Neutrophil Migration by Systemic Vitamin C in Neonates.” Journal of Perinatology 10, no. 2: 134–136. 2358895

[fsn370299-bib-0043] Wang, X. , and Z. Cheng . 2020. “Cross‐Sectional Studies: Strengths, Weaknesses, and Recommendations.” Chest 158, no. 1s: S65–S71. 32658654 10.1016/j.chest.2020.03.012

[fsn370299-bib-0044] Wu, X. , C. Wang , H. Li , et al. 2021. “Circulating White Blood Cells and Lung Function Impairment: The Observational Studies and Mendelian Randomization Analysis.” Annals of Medicine 53, no. 1: 1118–1128. 34259107 10.1080/07853890.2021.1948603 PMC8280897

[fsn370299-bib-0045] Wynn, T. A. 2011. “Integrating Mechanisms of Pulmonary Fibrosis.” Journal of Experimental Medicine 208, no. 7: 1339–1350. 21727191 10.1084/jem.20110551 PMC3136685

[fsn370299-bib-0046] Zeig‐Owens, R. , A. Singh , T. K. Aldrich , et al. 2018. “Blood Leukocyte Concentrations, FEV(1) Decline, and Airflow Limitation. A 15‐Year Longitudinal Study of World Trade Center‐Exposed Firefighters.” Annals of the American Thoracic Society 15, no. 2: 173–183. 29099614 10.1513/AnnalsATS.201703-276OC PMC5802620

[fsn370299-bib-0047] Zipf, G. , M. Chiappa , K. S. Porter , et al. 2013. “National Health and Nutrition Examination Survey: Plan and Operations, 1999–2010.” Vital Health Statistics 1 56: 1–37. 25078429

