# Supplementary material for: Dietary Vitamin C Intake Affects Lung Function Through White Blood Cell
Source: Food Sci Nutr. 2025 May 14;13(5):e70299. doi: 10.1002/fsn3.70299 (PMC12076004; doi:10.1002/fsn3.70299)
Supplement: Supplementary file 1 — Table S1. Selection and criteria of instrumental variables. [file FSN3-13-e70299-s001.docx]

| Exposure | Exposure id | P/ threshold | Clump/threshold | Outcome | Outcome id |
| --- | --- | --- | --- | --- | --- |
| Vitamin C intake | ukb-b-19390 | 5e-06 | 0.01/10000 | FEV1 | ebi-a-GCST007432 |
| Vitamin C intake | ukb-b-19390 | 5e-06 | 0.01/10000 | FVC | ebi-a-GCST90029027 |
| WBC | ebi-a-GCST90002374 | 5e-06 | 0.01/10000 | FVC | ebi-a-GCST90029027 |
| WBC | ebi-a-GCST90029003 | 5e-06 | 0.01/10000 | FEV1 | ebi-a-GCST007432 |

Table S1. Selection and criteria of instrumental variables
